# Web Comparison of Three Contingent Valuation Techniques in Women of Childbearing Age: The Case of Ovulation Induction in Quebec

**DOI:** 10.2196/13355

**Published:** 2020-02-06

**Authors:** Aissata Dieng, Jie He, Thomas G Poder

**Affiliations:** 1 University of Sherbrooke Deptartment of Economics Sherbrooke, QC Canada; 2 University of Montréal School of Public Health Montréal, QC Canada; 3 Centre de recherche de l’Institut universitaire en santé mentale de Montréal Montréal, QC Canada

**Keywords:** contingent valuation, elicitation technique, dichotomous choice, multiple-bounded discrete choice, willingness to pay, failed ovulation, ovulation induction

## Abstract

**Background:**

In Canada, 11.5% to 15.7% of couples suffer from infertility. Anovulation, or failed ovulation, is one of the main causes of infertility in women. In Quebec, the treatment for ovulation induction and other services related to assisted reproductive technology (ART) have been partially reimbursed by the government since 2010.

**Objective:**

This study aimed to compare the willingness to pay (WTP) of women of childbearing age to receive drug treatment in the event of failed ovulation according to 3 different contingent valuation methods.

**Methods:**

The following elicitation techniques were used: simple bid price dichotomous choice (DC), followed by an open-ended question (DC-OE), and a simplified multiple-bounded discrete choice (MBDC). Each participant was randomly assigned to 1 of 3 elicitation techniques. Bid prices ranged from Can $200 to Can $5000. Of the 7 bid prices, 1 was randomly proposed to each participant in the DC and DC-OE groups. For the DC-OE group, if the answer to the DC bid price was *no*, respondents were asked what was the maximum amount they were willing to pay. For the MBDC group, each respondent was offered an initial bid price of Can $1500, and the subsequent bid price offer increased or decreased according to the answer provided. “*Do not know*” responses were considered as a “*no*”, and each individual was questioned as to their certainty after each choice. WT*P* values were estimated using probit and bivariate models; the Welsh and Poe model was also performed for the MBDC group.

**Results:**

The survey was conducted from 2009 to 2010 with a total sample of 680 women. Analyses were performed on 610 respondents (199 DC, 230 DC-OE, and 181 MBDC). Of the 70 respondents who were excluded, 6 did not meet the age criterion, 45 had an annual income less than Can $2500, and 19 did not respond to the WTP question. Mean WT*P* values were Can $4033.26, Can $1857.90, and Can $1630.63 for DC, DC-OE, and MBDC, respectively. The WTP for MBDC “*definitely yes*” and “*probably yes*” values were Can $1516.73 and Can $1871.22, respectively. The 3 elicitation techniques provided WT*P* value differences that were statistically significant (*P*<.01). The MBDC was the most accurate method, with a lower confidence interval (Can $557) and a lower (CI/mean) ratio (0.34).

**Conclusions:**

A positive WTP for ovulation induction was found in Quebec. Adding a follow-up question resulted in more accurate WT*P* values. The MBDC technique provided a more accurate estimate of the WTP with a smaller and, therefore, more efficient confidence interval. To help decision making and improve the effectiveness of the fiscal policy related to the ART program, the WT*P* value elicited with the MBDC technique should be used.

## Introduction

### Background

According to the World Health Organization, infertility is defined as the inability to conceive after 12 months of unprotected sex [1]. In the epidemiology of infertile couples, Brzakowski et al [2] state that failed ovulation affects a large number of couples around the world—approximately 80 million people or 1 in 10 couples. In Canada, 11.5% to 15.7% of couples suffer from infertility, according to the Institut national d’excellence en santé et en services sociaux [3]. According to the Association of Gynecologists and Obstetricians of Quebec, there is a decrease in fertility in 84% of couples, including 10% of infertility in women (eg, fallopian tube blockage on both sides) and 6% infertility in men (eg, no spermatozoa) [4]. Anovulation or abnormality of ovulation is one of the main causes of infertility in women. To counteract this issue, a drug treatment that aims to induce ovulation is needed. In Quebec, this treatment and other services of assisted reproductive technology (ART) have been partially reimbursed by the government since 2010 [1].

The benefit of the drug treatment to induce ovulation in adult female patients with infertility is generally measured by the proportion of women who ovulate as a result of such treatment [5]. It is difficult to measure the monetary benefit of this treatment without exchange value, which implies the problem of comparing the costs of this treatment with its *benefits*. One way to obtain a monetary value of this benefit is to estimate women’s willingness to pay (WTP) for this infertility treatment.

Several methods can be used to estimate this monetary value, including the contingent valuation method (CVM) [6,7]. This method is increasingly used in health economics, as it provides a monetary value for nonmarket goods and services using a fictitious market [8]. The CVM consists in asking a hypothetical question using a variety of survey techniques (eg, telephone, face to face, internet, and postal mail) to measure the maximum amount that individuals would be willing to pay for something (in this case, the fertility treatment) and its consequent effects. This method offers several elicitation techniques that correspond to different ways of formulating the WTP question.

Although different variants exist, the 4 main techniques reported in the literature are bidding game (BG), payment card (PC), open-ended (OE) questions, and dichotomous choice (DC) [9,10]. The BG is the oldest elicitation technique [6]. The respondent is randomly assigned a particular bid from a range of predetermined bids. The respondent is then asked to say *yes* or *no* to that particular bid, and the process continues until the highest positive response is recorded [11]. The PC consists of presenting the respondent with a series of offers in a table in which the individual circles the amount corresponding to his or her WTP [12]. The OE consists in asking the respondent directly what is the maximum amount he or she would be willing to pay for a given good or service [12,13]. In the DC approach, the respondent only answers *yes* or *no* to a given amount.

### Objective

In this study, 3 CVMs were compared: DC, DC followed by an OE question (DC-OE), and a simplified multiple-bounded discrete choice (MBDC), which is very similar to a BG. These 3 methods were chosen because of their simplicity and because they are widely used in the literature. The main objective of this study was to assess the WTP of women of childbearing age to receive a drug treatment in the event of failed ovulation according to the 3 different CVMs. More specifically, this study aimed to assess whether these 3 techniques generate statistically different WTPs and, if so, to determine which method is the most accurate.

## Methods

### Study Design and Population

The data used in this study were from a survey conducted in Quebec between January 2009 and February 2010. Inclusion criteria required participants to be a woman aged 18 to 45 years and to agree to complete the survey in French. Women were excluded if they had an annual income less than or equal to Can $2500 (this amount corresponded to the middle of the lowest bracket proposed for annual income and because it is unlikely that women can afford an infertility treatment with this income) or if they did not respond to the WTP question. No sample size was calculated, but 200 patients per elicitation method were targeted, which is the usual number for this type of study [14].

### Data Collection

The data were collected through a Web survey, which were first distributed using an email listing from previous studies (ie, respondents from previous studies who accepted to be contacted for future research) and were then distributed by a Web survey company. Participants were randomly allocated to 1 of the 3 elicitation methods tested (DC, DC-OE, or MBDC).

### Questionnaire

Each questionnaire had 3 main components: introduction, socioeconomic variables, and WTP questions. The introduction presented a definition of infertility, gave the prevalence of infertility (including infertility related to ovulation failure), the type of treatment associated, the probability of success, and the associated risks. The socioeconomic variables included age (years), weight (kilograms), height (centimeters), employment status, is the job stressful (yes or no), individual annual income (using brackets), educational level, civil status, number of children, smoking (yes or no), general health (5 levels), fertility problems (yes or no), actually pregnant (yes or no), desire for a child (yes or no), and a ranking of 10 items (eg, have good health, have children, be financially comfortable).

The third component was the WTP question about receiving ovulation failure treatment, along with another question about the degree of certainty of the respondent’s answer. For DC and DC-OE, 7 price levels were randomly assigned to different versions of the questionnaire (Can $200, Can $500, Can $1000, Can $1500, Can $2000, Can $3000, and Can $5000). For the DC-OE, if the answer was *no* or *do not know*, respondents were asked to report the maximum amount they were willing to pay for this service. For the MBDC, the respondent was offered an initial WTP amount of Can $1500, where the possible answers were *yes*, *no*, or *do not know*. If the first answer was positive, the price increased to Can $3000; if it was negative or *do not know*, the price decreased to Can $500. Unlike a traditional MBDC, which uses a random start price, our approach used a predetermined starting price of Can $1500, and only 3 bids were possible to more quickly end the round of questioning. For all CVM, the *do not know* response was considered as a *no*, and a question about the certainty of the answer was asked to individuals after each choice (not at all certain, not certain, more or less certain, certain, and quite certain).

### Data Analysis

Overall, 2 comparison criteria were used to judge the accuracy of the estimates. The obtained estimated WTP and standard deviations were compared between the 3 subsamples. The efficiency of the estimates was measured with the ratio of confidence interval on mean WTP. The expected efficiency associated with a follow-up WTP question is based on the fact that the confidence intervals should be narrower and closer to the mean WT*P* value [15]. Multivariate probit models were computed to estimate WTP for each method in considering only the *yes* or *no* responses. For the DC-OE, a bivariate probit model was used with the *yes* or *no* response and the OE response as the dependent variable. For MBDC, the Welsh and Poe model [15] was computed for *definitely yes*, *probably yes*, and *do not know* responses.

To calculate the mean WT*P* value of the probit models, the coefficients of each variable were multiplied by the mean value of the total sample and divided by the bid coefficient and the bid mean (thus, WTP equals ∑(β_j_*µ_j_) divided by (β_bid_*µ_bid_), where β_j_=coefficient of variable j and µ_j_=mean of variable j). Considering the mean value of the total sample allowed to better consider differences in WT*P* values associated with the 3 elicitation techniques and to reduce the effect of socioeconomic differences observed in the subsamples. For the bivariate model, the WTP was directly obtained by multiplying the coefficients of the equation by the mean value of each variable, again with the mean value of the total sample. WTP was also estimated using the parametric bootstrap method developed by Krinsky and Robb [16], with 1000 repetitions. This method consists of making a large number of draws from a multivariate normal distribution with the means and the variance-covariance matrix of the estimated parameters. The different simulated WT*P* values were calculated from the joint distribution of the coefficients. This method gives precise confidence intervals. Details of the WTP estimation methods are presented in Multimedia Appendix 1 [17-20].

## Results

### Patient Characteristics

**Figure 1:**
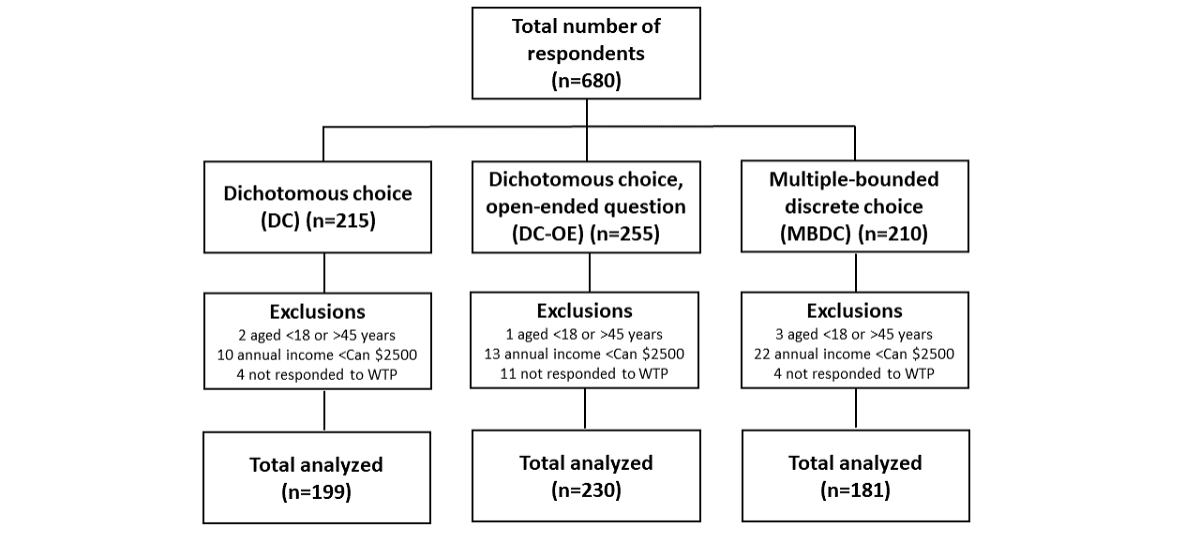
Flowchart of respondents randomly distributed among the 3 elicited methods (dichotomous choice, dichotomous choice followed by an open-ended question, or multiple-bounded discrete choice).

The total sample consisted of 680 women; of these women, 215 responded to the DC, 255 responded to the DC-OE, and 210 responded to the MBDC (Figure 1). A total of 70 respondents were excluded: 6 did not meet the age criterion, 45 had an annual income less than Can $2500, and 19 did not respond to the WTP question. Analyses were thus conducted on 610 respondents (199 DC, 230 DC-OE, and 181 MBDC). Although the CVM groups were randomly distributed, they differed significantly for a number of variables. The respondents answering the DC questionnaire were older, had higher annual income, and were in better health. Those answering the DC-OE questionnaire had fewer problems with infertility, but among those, the percentage of failed ovulation was higher. The people invited to answer the MBDC questionnaire had a lower educational level, were mostly smokers, had less stress, and were less often employed (Table 1).

**Table 1:** Descriptive statistics.

Variable			Dichotomous choice (n=199)		Dichotomous choice followed by an open-ended question (n=230)		Multiple-bounded discrete choice (n=181)
Age (years), mean (range)			32 (18-45)		30 (18-45)^a^		30 (18-45)^a^
Annual income (Can $), mean (range)			42,622 (7500-130,000)		37,615 (7500-130,000)^b^		34,475 (7452-130,000)^a,c^
**Schooling, n (%)**							
	Secondary school	31 (15.6)		41 (17.8)		42 (23.2)^b,^^d^	
	College	78 (39.2)		93 (40.4)		63 (34.8)	
	University	90 (45.2)		96 (41.7)		76 (42.0)	
Very good health, n (%)			58 (29.2)		43 (18.7)^a^		36 (19.9)^a^
Current smoker, n (%)			39 (19.6)		45 (19.6)		51 (28.2)^a,e^
Stressful job, n (%)			174 (87.4)		198 (86.1)^a^		145 (80.1)^c^
Ovulation failure, n (%)			7 (3.5)		14 (6.1)^a^		5 (2.8)^e^
Infertility problem, n (%)			26 (13.1)		25 (10.9)^a^		22 (12.2)^e^
Having a child is important, n (%)			125 (62.8)		147 (63.9)		108 (59.7)
Employee, n (%)			158 (79.4)		183 (79.6)		130 (71.8)^b,e^

^a^*P*<.01 (comparison of dichotomous choice versus dichotomous choice followed by an open-ended question and dichotomous choice versus multiple-bounded discrete choice).

^b^*P*<.05 (comparison of dichotomous choice versus dichotomous choice followed by an open-ended question and dichotomous choice versus multiple-bounded discrete choice).

^c^*P*<.05 (comparison of dichotomous choice followed by an open-ended question versus multiple-bounded discrete choice).

^d^*P*<.1 (comparison of dichotomous choice followed by an open-ended question versus multiple-bounded discrete choice).

^e^*P*<.01 (comparison of dichotomous choice followed by an open-ended question versus multiple-bounded discrete choice).

### Responses to Willingness to Pay Questions

**Figure 2:**
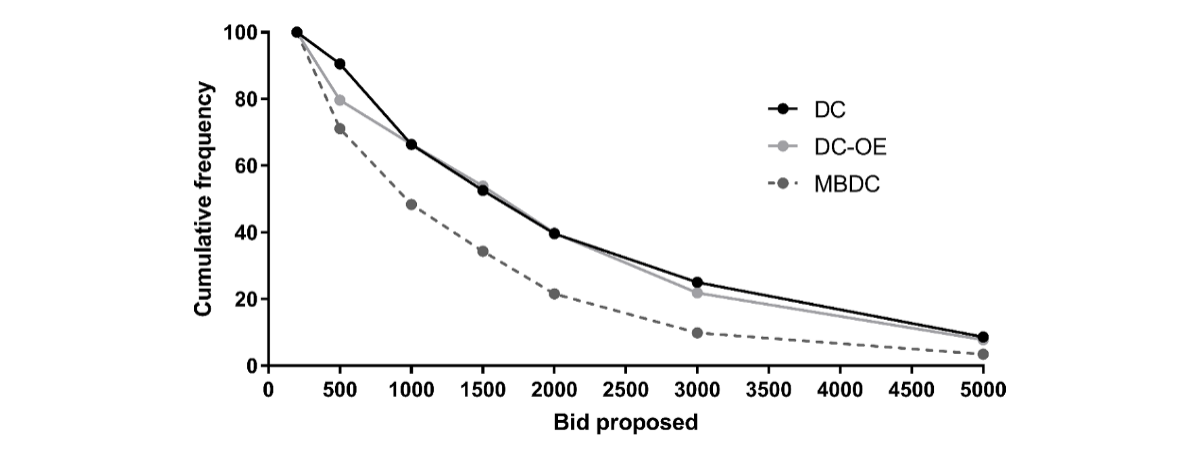
Cumulative decreasing frequencies of positive answers yes. DC: dichotomous choice; DC-OE: dichotomous choice followed by an open-ended question; MBDC: multiple-bounded discrete choice.

As we expected, the higher the offered bid price, the lower the proportion of *yes* answers. The percentage of *yes* answers for the lowest value (Can $200) was 86% (12/14) for DC, 77% for DC-OE (26/34), and 100.0% (181/181) for MBDC. For the highest price (Can $5000), the percentage of *yes* answers was 26% (10/39) for DC, 53% (10/19) for DC-OE, and 12.7% (23/181) for MBDC. It should be noted that the decrease was gradual with DC and MBDC but more stable with DC-OE. When the cumulative decreasing frequencies of positive responses were analyzed, the DC and DC-OE had similar distributions, but the MBDC decreased more rapidly (Figure 2).

### Willingness to Pay Estimated With Dichotomous Choice

The results of the probit analysis are presented in Table 2. The higher the offer (bid), the lower the probability to say yes to the WTP question (*P*<.01). The contribution of each explanatory variable to the WTP was calculated with the ratio of coefficients (−β_variable_/β_bid_). For example, women with a university education were willing to pay Can $2338 (−.788/−.000337) more than women without a university education (*P*<.01). Women with very good health, with a stressful job, or who considered having a child to be important were also willing to pay more (Can $1772, *P*<.05; Can $593, *P*<.10; and Can $1395, *P*<.05, respectively).

**Table 2:** Estimation results with probit and bivariate analysis.

Variables	Dichotomous choice		Dichotomous choice followed by an open-ended question					Multiple-bounded discrete choice	
	Probit (R^2^=0.2375; N=199)		Probit (R^2^=0.0863; N=230)		Bivariate (R^2^=0.0633; N=230)			Probit (R^2^=0.2807; N=1245)	
	Coefficient	*T* test (187)	Coefficient	*T* test (218)	Coefficient	*T* test (218)	Coefficient		*T* test (1233)
Bid	−*0.000337*^a^	−*4.71*^b^	−*0.000111*	−*1.67*^c^	—^d^	—	−*0.000573*		−*17.17*^b^
Age (years)	−0.0264	−1.54	−0.0171	−1.15	−11.50	−0.52	−*0.0279*		−*4.43*^b^
Annual income	0.00000310	0.69	0.00000509	1.00	0.00749	0.97	*0.00000395*		*1.77* ^c^
University	*0.788*	*3.34* ^b^	*0.423*	*2.08* ^e^	*523.9*	*1.69* ^c^	*0.343*		*3.61* ^b^
Very good health	*0.597*	*2.42* ^e^	−0.0571	−0.24	−110.2	−0.31	0.0157		0.15
Infertility problem	−0.165	−0.43	−0.113	−1.07	−170.3	−1.08	−0.202		−1.36
Ovulation failure	0.728	1.03	0.0395	0.29	−34.94	−0.17	0.384		1.39
Current smoker	0.401	1.49	−0.388	−1.62	−*602.2*	−*1.72*^c^	*0.422*		*4.53* ^b^
Stressful job	*0.200*	*1.81* ^c^	−0.0770	−0.30	83.55	0.21	−0.0546		−0.49
Having a child is important	*0.470*	*2.1* ^e^	0.312	1.63	209.1	0.73	*0.554*		*6.40* ^b^
Employee	0.421	1.63	0.242	0.99	207.8	0.58	*0.185*		*1.88* ^c^
Constant	0.149	0.25	0.280	0.48	*2218.5*	*2.60* ^b^	*0.458*		*1.74* ^c^
Sigma	—	—	—	—	*1583.9*	*13.34* ^b^	—		—

^a^Italics indicate that the term is statistically significant.

^b^*P*<.01.

^c^*P*<.1.

^d^Not applicable.

^e^*P*<.05.

### Willingness to Pay Estimated With Dichotomous Choice Followed by an Open-Ended Question

The coefficients of the explanatory variables of the bivariate model directly illustrate women’s WTP. For DC-OE, when the offer (bid) was higher, the WTP was significantly lower (*P*<.1). Women with a university education were willing to pay Can $3811 more in the probit model (*P*<.05) and Can $524 more in the bivariate model (*P*<.1). Women who smoked were willing to pay Can $602 less than other women (*P*<.1).

### Willingness to Pay Estimated With Multiple-Bounded Discrete Choice

The probit model results are presented in Table 2, and the results using the model by Welsh and Poe are presented in Table 3. As for the DC and DC-OE methods, if the offer (bid) was higher, the WTP was significantly lower (*P*<.01). The probit model for MBDC had more significant variables that explain the WTP. Older women were willing to pay Can $48.69 (*P*<.01) and Can $82.50 (*P*<.1) less than other women per additional year in the probit model and in the *probably yes* model by Welsh and Poe, respectively. Women with higher incomes were willing to pay more than other women (*P*<.1) in the probit model. Women with a university education were willing to pay Can $599 more in the probit model (*P*<.01) and Can $1058 more in the *probably yes* model (*P*<.1). Women who smoked were willing to pay Can $736 (*P*<.01) more in the probit model and Can $941 (*P*<.01) more in the *do not know* model. Women for whom having a child was a priority were willing to pay more than others at an amount of Can $967 (*P*<.01) and Can $581 (*P*<.1) in the probit and *do not know* models, respectively. Having a job increased women’s WTP by Can $323 compared with other women in the probit model.

**Table 3:** Estimation results using the model by Welsh and Poe.

Variables		Definitely yes (N=181)^a^		Probably yes (N=181)^a^			Do not know (N=181)^a^		
		Estimate	*T* test (169)		Estimate	*T* test (169)		Estimate	*T* test (169)
**Equation 1**									
	Constant	−*0.000626*^b^	−*14.08*^c^		−*0.000343*	−*9.68*^c^		−*0.000542*	−*12.56*^c^
**Equation 2**									
	Age (years)	−0.00412	−0.31		−*0.0283*	−*2.13*^d^		−0.0133	−1.05
	Annual income	0.00000230	0.48		0.00000126	0.27		0.00000130	0.28
	University	0.271	1.42		*0.363*	*1.86* ^d^		0.280	1.44
	Very good health	0.0331	0.16		0.0661	0.30		−0.115	−0.54
	Infertility problem	−0.175	−0.59		−0.192	−0.62		−0.130	−0.41
	Ovulation failure	0.460	0.74		0.309	0.51		0.135	0.23
	Current smoker	0.213	1.12		0.218	1.12		*0.510*	*2.66* ^c^
	Stressful job	−0.0288	−0.12		0.177	0.74		−0.175	−0.74
	Having a child is important	0.128	0.72		0.279	1.54		*0.315*	*1.76* ^e^
	Employee	−0.156	−0.77		−0.0866	−0.42		0.0772	0.38
	Constant	0.558	1.01		0.778	1.41		*1.155*	*2.16* ^d^

^a^*P* value<.001.

^b^Italics indicate that the term is statistically significant.

^c^*P*<.01.

^d^*P*<.05.

^e^*P*<.1.

### Mean Willingness to Pay Estimated

Table 4 reports the mean WTP for each subsample and their confident intervals obtained with Krinsky and Robb’s [16] bootstrap method. As shown, women were, in general, willing to pay for an ovulation failure treatment an average of Can $4033.26 in the DC questionnaire, Can $1857.90 in the DC-OE questionnaire, and Can $1630.63 in the MBDC questionnaire. The mean WTPs for MBDC definitely yes and probably yes were Can $1516.73 and Can $1871.22, respectively. A Student t test revealed a statistically significant difference among the mean WTPs obtained from the DC, DC-OE, and MBDC subsamples (all *P*<.01). The MBDC method can be considered the most accurate, with the lowest confidence interval (896.51) and the lowest (CI/mean) ratio (0.53). The DC-OE method had a confidence interval higher than MBDC and a CI/mean ratio of 1.04. The least accurate approach was the DC method.

Comparing DC with DC-OE, we can see that adding 1 more question after the DC WTP question improves the accuracy of the WTP estimates. However, our results also revealed the existence of an anchoring effect in the DC-OE approach, where the implicit WT*P* values of the respondents were influenced by the first proposed bid price. With the Herriges and Shogren’s model [21], the gamma coefficient (SE) was 0.7402 (0.0166), and the 95% CI was from 0.7074 to 0.7729 (*P*<.001).

**Table 4:** Mean willingness to pay estimated with probit, bivariate, or Krinsky and Robb methods.

Method		Average WTP^a^		Mean difference (95% CI)		CI/WTP	
**Probit and bivariate methods**							
	DC^b^ (N=199)		4033.26		4386.01 (1840.25 to 6226.26)		1.09
	DC-OE^c^ (N=230)		1857.90		1945.1 (885.35 to 2830.45)		1.05
	MBDC^d^—probit (N=1245)		1630.63		556.64 (1352.31 to 1908.95)		0.34
	MBDC—definitely yes (N=181)		1516.73		2796.86 (118.30 to 2915.16)		1.84
	MBDC—probably yes (N=181)		1871.22		4926.29 (−591.92 to 4334.37)		2.63
	MBDC—do not know (N=181)		2514.49		3032.39 (998.29 to 4030.68)		1.21
**Krinsky and Robb’s methods**							
	DC (N=199)		4750.18		6985.57 (3911.67 to 10,897.24)		1.47
	DC-OE (N=230)		1857.90		1924.93 (895.44 to 2820.37)		1.04
	MBDC—probit (N=1245)		1701.37		896.51 (1103.43 to 1999.94)		0.53

^a^WTP: willingness to pay.

^b^DC: dichotomous choice.

^c^DC-OE: dichotomous choice followed by an open-ended question.

^d^MBDC: multiple-bounded discrete choice.

## Discussion

### Principal Findings

A total of 3 elicitation techniques were used to assess women’s WTP for an ovulation induction treatment in case of failed ovulation. One of the main objectives was to discover whether a significant difference exists between different WTP elicitation approaches.

The results show that the DC technique yielded higher estimated WTP than the other 2 techniques. The higher value for WTP with DC methods is consistent with the literature [22-26]. In Welsh and Poe’s study [26], they concluded that the WTP obtained by the DC technique was higher than that obtained by the *not sure* model. In our study, we also find that the DC WTP was statistically larger than the Welsh and Poe *not sure* model, and the DC-OE WTP did not statistically differ from the *probably yes* model of Welsh and Poe. On the contrary, the WTP of the MBDC method was between the *definitely yes* and the *probably yes* models of Welsh and Poe. The comparison of DC and DC-OE was also consistent with the findings of Hanneman et al [15], who used a bivariate model to compare estimates of DC and double-bounded DC. They found that the double-bounded model reduced the variance of the estimated parameters and decreased the covariance terms. They concluded that the double-bounded DC model was more efficient after correcting for the anchoring effect.

The value added by a follow-up question is based on the fact that the confidence intervals are closer to the estimated WTP and that the latter is, therefore, more accurate [15]. This is the case in our study, where the WTP estimate with the DC-OE model was more accurate than the DC approach. By comparing the confidence intervals and standard deviations of the different techniques, our results show that the MBDC technique gave lower mean WTPs and smaller standard deviations than the other 2 techniques. Therefore, based on efficiency as the criterion of comparison (ie, the ratio of the confidence interval to the mean WTP [16]), the MBDC technique is preferable. The DC-OE gave a confidence interval that was wider than that of MBDC but still lower than that of DC. Our results are similar to the study by Scarpa and Bateman [27], where the authors concluded that MBDC WTPs are more efficient and that including one additional question in a contingent valuation survey improves the effectiveness of the WTP, although biases caused by a potential anchoring effect are likely to occur.

The estimated WTP in our study shows dissimilar results to the study by Poder et al [5] about failed ovulation. In their study, they found that the mean WTP for a medical treatment for ovulation induction was Can $3400 CAD in the DC technique, where *do not know* answers were considered as a *no*. We found a higher WTP in our DC database (Can $4033). This difference may be because of the mode of collection they used (paper and Web), their higher number of observations (327 vs 215 subjects), or the sociodemographic characteristics of their sample. However, what is consistent in these studies is that women have a positive WTP for infertility treatment. In our specific study about ovulation induction, the standard treatment is to administer clomiphene citrate over a 6-month period. This specific treatment can be done at a very low cost (less than Can $500) when compared with the WT*P* value found. This indicates that the social value of infertility treatment is highly valued by women and that to invest in it is worth it.

Our study gave coefficients of the expected signs, although the positive coefficient of the variable *income* was not significant in the 3 techniques. This result suggests that women’s responses were independent of their income. One explanation for this is that infertility is of major importance in their lives, regardless of income. A similar result was found in the study by Poder et al [5]. Moreover, the negative coefficient on the variable of age implies that older women place less importance on care for ovulation failure, perhaps because women’s fertility decreases with age. The results of our different regressions also concur with the predictions of economic empirical theories, which state that women’s WTP decreases with age [28,29].

This study has a number of limitations, so the results should be interpreted with caution. As we used a convenience sample, we cannot say with certainty that our regression equations will give the same results if applied to a larger or different sample because of the lack of representativeness. Another limitation of this study is that our approach used a fixed predetermined starting price of Can $1500 in the MBDC technique. This choice may have led to an anchoring effect, as individuals focus on the first proposition (Can $1500), and thus, their answers to the second and third questions may be influenced by the first bid offered. Unlike other techniques (DC and DC-OE) that use random starting prices between Can $200 and Can $5000, this anchoring effect cannot be assessed in the MBDC.

Each of the 3 elicitation techniques has its disadvantages. The DC technique yielded higher estimated WTP with little WTP information (ie, only 1 WTP question, so we only know if their maximum WTP is higher or lower to the bid proposed). However, the DC technique is more similar to the real market situation of *take it or leave it* [22]. Although the DC technique followed by an OE question provides more information for those answering *no* or *do not know*, it does not add information for those answering *yes* to the first question (ie, we do not know the maximum offer that would be accepted); moreover, the responses to the second question can introduce the possibility of strategic behavior on the part of respondents. Respondents may feel that giving a positive WTP to the second question may allow the government to increase their claims but answering *zero* to the second question could be because of the impression that the quality of the service offered may be reduced. Furthermore, a high zero-value rate (ie, many zeros) and an anchoring effect occur in the DC-OE technique.

### Conclusions

In their study on psychosocial services for couples in infertility treatment, Read et al [30] reported that infertility is associated with considerable distress, and treatment is often characterized by cycles of hope and disappointment. Regardless of age, failed ovulation is the most common cause of infertility in women; today, it can be treated with fertility drugs [5]. In this study, the goal was to test whether an elicitation technique may have an effect on the estimation of WTP for women of childbearing age for a failed ovulation treatment service. The data from the 3 techniques reveal that women with a higher level of education placed more importance on the treatment of failed ovulation than other women. We also note that in the MBDC technique, the lowest bid price offered (Can $200) was accepted by all respondents. Thus, infertility treatment is seen as having a positive value.

We also compared the mean WTPs of the different techniques and found significant differences among the estimated WTPs. Adding a follow-up question resulted in more accurate WTPs but created anchoring biases. Results also indicated that the simplified MBDC technique provided more accurate estimates of the WTP with a smaller and, therefore, more efficient confidence interval. Consequently, for the purpose of a more efficient fiscal policy, the simplified MBDC technique provided the most appropriate WT*P* value.

## Appendix

Multimedia Appendix 1 Estimates of willingness to pay.

## Abbreviations

ART: assisted reproductive technology
BG: bidding game
CVM: contingent valuation method
DC: dichotomous choice
DC-OE: dichotomous choice followed by an open-ended question
MBDC: multiple-bounded discrete choice
PC: payment card
OE: open-ended
WTP: willingness to pay

## References

[1] Zegers-Hochschild F, Adamson GD, de Mouzon J, Ishihara O, Mansour R, Nygren K, Sullivan E, Vanderpoel S, International Committee for Monitoring Assisted Reproductive Technology, World Health Organization (2009). International Committee for Monitoring Assisted Reproductive Technology (ICMART) and the World Health Organization (WHO) revised glossary of ART terminology, 2009. Fertil Steril.

[2] Brzakowski M, Lourdel E, Cabry R, Oliéric M, Claeys C, Devaux A, Copin H, Merviel P (2009). Epidemiology of the infertile couple. J Obstet Gynecol Reprod Biol.

[3] Institut national d'excellence en santé et en services sociaux (INESSS) (2015). Assisted Human Reproduction - Medical Criteria for Eligibility for Publicly Funded Treatments and Safety of Repetition in In Vitro Fertilization Cycles.

[4] AOGQ: Association of Obstetricians and Gynecologists of Quebec.

[5] Poder TG, He J, Simard C, Pasquier JC (2014). Willingness to pay for ovulation induction treatment in case of WHO II anovulation: a study using the contingent valuation method. Patient Prefer Adherence.

[6] Mitchell RC, Carson RT (1989). Using Surveys to Value Public Goods: The Contingent Valuation Method.

[7] Hanemann WM (1994). Valuing the environment through contingent valuation. J Econ Perspect.

[8] Ryan M, Scott DA, Donaldson C (2004). Valuing health care using willingness to pay: a comparison of the payment card and dichotomous choice methods. J Health Econ.

[9] Boyle KJ, Johnson FR, McCollum DW, Desvousges WH, Dunford RW, Hudson SP (1996). Valuing public goods: discrete versus continuous contingent-valuation responses. Land Econ.

[10] Venkatachalam L (2004). The contingent valuation method: a review. Environ Impact Assess Rev.

[11] Randall A, Ives B, Eastman C (1974). Bidding games for valuation of aesthetic environmental improvements. J Environ Econ Manage.

[12] Reaves D, Kramer R, Holmes T (1999). Does question format matter? Valuing an endangered species. Environ Resour Econ.

[13] Walsh RG, Loomis JB, Gillman RA (1984). Valuing option, existence, and bequest demands for wilderness. Land Econ.

[14] Lin P, Cangelosi MJ, Lee DW, Neumann PJ (2013). Willingness to pay for diagnostic technologies: a review of the contingent valuation literature. Value Health.

[15] Hanemann M, Loomis J, Kanninen B (1991). Statistical efficiency of double-bounded dichotomous choice contingent valuation. Am J Agric Econ.

[16] Krinsky I, Robb AL (1986). On approximating the statistical properties of elasticities. Rev Econ Stat.

[17] Alberini A, Kanninen B, Carson RT (1997). Modeling response incentive effects in dichotomous choice contingent valuation data. Land Econ.

[18] Carson RT, Flores NE, Martin KM, Wright JL (1996). Contingent valuation and revealed preference methodologies: comparing the estimates for quasi-public goods. Land Econ.

[19] Cameron TA, Huppert DD (1989). OLS versus ML estimation of non-market resource values with payment card interval data. J Environ Econ Manage.

[20] Wang H, He J (2011). Estimating individual valuation distributions with multiple bounded discrete choice data. Appl Econ.

[21] Herriges JA, Shogren JF (1996). Starting point bias in dichotomous choice valuation with follow-up questioning. J Environ Econ Manage.

[22] Johannesson M, Jönsson B (1991). Economic evaluation in health care: is there a role for cost-benefit analysis?. Health Policy.

[23] Seller C, Stoll JR, Chavas JP (1985). Validation of empirical measures of welfare change: a comparison of nonmarket techniques. Land Econ.

[24] Johnson RL, Bregenzer NS, Shelby B, Johnson RL, Johnson GV (1990). Contingent valuation question Foryats: dichotomous choice versus open-ended responses. Economic Valuation Of Natural Resources: Issues, Theory, And Applications.

[25] Desvousges WH, Johnson FR, Dunford RW, Boyle KJ, Hudson SP, Wilson KN, Hausman JA (1993). Chapter III - measuring natural resource damages with contingent valuation: tests of validity and reliability. Contributions to Economic Analysis.

[26] Welsh MP, Poe GL (1998). Elicitation effects in contingent valuation: comparisons to a multiple bounded discrete choice approach. J Environ Econ Manage.

[27] Scarpa R, Bateman I (2000). Efficiency gains afforded by improved bid design versus follow-up valuation questions in discrete-choice CV studies. Land Econ.

[28] Alberini A, Cropper M, Krupnick A, Simon NB (2004). Does the value of a statistical life vary with age and health status? Evidence from the US and Canada. J Environ Econ Manage.

[29] Carson RT, Flores NA (2000). eScholarship.

[30] Read SC, Carrier ME, Boucher ME, Whitley R, Bond S, Zelkowitz P (2014). Psychosocial services for couples in infertility treatment: what do couples really want?. Patient Educ Couns.

