# Supplementary material for: Web Comparison of Three Contingent Valuation Techniques in Women of Childbearing Age: The Case of Ovulation Induction in Quebec
Source: Interact J Med Res. 2020 Feb 6;9(1):e13355. doi: 10.2196/13355 (PMC7055751; doi:10.2196/13355)
Supplement: Multimedia Appendix 1 [file ijmr_v9i1e13355_app1.docx]

APPENDIX

# Estimates of willingness to pay

### Dichotomous choice method

For DC, the respondent answered “yes”, “no” or “do not know”. If the response was yes, the function to be estimated is: ${WTP}_{i}=\mu+X_{i}\beta+\varepsilon_{i}>B_{i}$, Where *B_i_:* proposed bid, μ: constant of the WTP, σ: variance of the WTP in the population, *X_i_*: characteristic vector of individual *i*. One respondent will say Yes if WTP_i_>=B_i_ and he/she will say No if WTP_i_<B_i._ We can write the probability of yes as:

$$Prob\left( {WTP}_{i}=yes | B_{i} \right)=Prob\left( {WTP}_{i}\geq B_{i} \right)$$

and the probability of “no” becomes:

$$Prob\left( {WTP}_{i}<B_{i} \right)=1-Prob\left( {WTP}_{i}\geq B_{i} \right)$$

The maximization function of the log-likelihood (L) is written as:

$$logL=\sum_{i=1}^{N} \left\{ \sum_{y\in oui} log\left[ Prob\left( {WTP}_{i}\geq B_{i} \right) \right]+\sum_{y\in non} log\left[ 1-Prob\left( {WTP}_{i}\geq B_{i} \right) \right] \right\}$$

### Dichotomous choice with open-ended question

For the DC-OE, the same question than DC was asked, but if the answer was “no” or “do not know”, respondents were asked what was the maximum amount they were willing to pay for this service. So each individual was in one of the following categories: “Yes” or “No-maximal amount”. The respondent answering “yes” has WTP_i_>B_i_. If she answered “no”, we have $A_{i}\leq{WTP}_{i}<B_{i}$.

The maximization function of the log-likelihood (L) can be be written as following, similar to a double-bounded model (Alberini et al. 1997):

$$logL=\sum_{i=1}^{N} \left\{ \sum_{y \in yes} log\left[ Prob\left( {WTP}_{i}\geq B_{i} \right) \right]+\sum_{y \in no\_\max amount} log\left[ Prob(A_{i}\leq{WTP}_{i,2}<B_{i}) \right] \right\}$$

With the DC-OE method, there is a risk of anchoring effect. This bias is present when the answers of the individuals for the second price proposed is influenced by the first proposed price. The double-bounded model assumes that the respondents' WTP does not vary between the two evaluations questions [26]. To capture the potential anchor effect, we follow the model proposed in Herriges and Shogren [27], where respondents’ WTP value after the first DC question (WTP_i,2_) is calculated as a weighted average between the initial WTP_i,1_ and the bid of the first question B_i_. So our estimation model after the modification proposed by Herriges and Shogren [27] can be written as follows:

${WTP}_{i, 2}=\left( 1-\gamma\right){WTP}_{i,1}+{\gamma B}_{i}$

$$logL=\sum_{i=1}^{N} \left\{ \sum_{y \in yes} log\left[ Prob\left( {WTP}_{i}\geq B_{i} \right) \right]+\sum_{y \in no\_\max amount} log\left[ Prob(A_{i}\leq\left( 1-\gamma\right){WTP}_{i,1}+{\gamma B}_{i}<B_{i}) \right] \right\}$$

With 0 ≤ γ ≤ 1 and measures the anchoring effect;

If γ = 0, *WTP_1_ = WTP_2_* and there was no anchoring bias. If γ > 0, *WTP_1_ ≠ WTP_2_* and there was an anchoring bias.

If γ = 1, there is the maximal anchoring bias and *WTP_2_ = bid_1_*

### Multiple-bounded discrete choice

For the MBDC, the respondent was offered an initial WTP amount, where the answers were “yes”, “no” or “do not know”. The “do not know” response was considered as a “no” as suggested by Carson et al. [28]. If the first answer was positive, a higher price was proposed subsequently, whereas for a negative response, a lower price was proposed. A question about the certainty of the answer was also asked to individuals after each choice to measure their degree of certainty (i.e., not at all certain, not certain, more or less certain, certain and, quite certain). To combine these two information, the WTP analysis was based on the multiple bounded maximum likelihood interval modelling approach was used [21]. This approach generalizes the double-bounded model’s maximum likelihood interval modelling approach used for payment card data in Cameron and Huppert [29]. This approach can be applied to positive choices that measure different levels of affirmation [30] i.e. "Definitely Yes", "Probably Yes", "Do not Know", "Probably No" and "Definitely No". This approach sets the lower bound, X_iL_, at the level of the highest proposed price for a specified certain degree of certainty such as ‘definitely yes’ or “probably yes”. The upper bound, X_iU_, was the next higher proposed price to which respondents gave a lower degree of certainty (i.e., the next bid price level to X_iL_) [21]. Welsh and Poe supposed that WTP_i_ should lie somewhere in the switching interval [X_iL_, X_iU_]. The probability for WTP_i_ falling between any two price thresholds is $\mathrm{Prob}\left( {{WTP}_{i}<X}_{iU} \right)- \mathrm{Prob}\left( {{WTP}_{i}>X}_{iL} \right)$, resulting in the corresponding log-likelihood function:

$$LogL=\sum_{i} log\left[ \mathrm{Prob}\left( {{WTP}_{i}<X}_{iU} \right)- \mathrm{Prob}\left( {{WTP}_{i}>X}_{iL} \right) \right]$$
